# Computational discovery and functional validation of novel fluoroquinolone resistance genes in public metagenomic data sets

**DOI:** 10.1186/s12864-017-4064-0

**Published:** 2017-09-02

**Authors:** Fredrik Boulund, Fanny Berglund, Carl-Fredrik Flach, Johan Bengtsson-Palme, Nachiket P. Marathe, DG Joakim Larsson, Erik Kristiansson

**Affiliations:** 1 Department of Mathematical sciences, Chalmers university of Technology and University of Gothenburg, Gothenburg, Sweden; 2 Centre for Antibiotic Resistance Research, University of Gothenburg, Gothenburg, Sweden; 3 Department of Infectious Diseases, Institute of Biomedicine, University of Gothenburg, Gothenburg, Sweden

**Keywords:** *Qnr*, Fluroquinolone resistance, Horizontal gene transfer, Hidden Markov models, Metagenomics

## Abstract

**Background:**

Fluoroquinolones are broad-spectrum antibiotics used to prevent and treat a wide range of bacterial infections. Plasmid-mediated *qnr* genes provide resistance to fluoroquinolones in many bacterial species and are increasingly encountered in clinical settings. Over the last decade, several families of *qnr* genes have been discovered and characterized, but their true prevalence and diversity still remain unclear. In particular, environmental and host-associated bacterial communities have been hypothesized to maintain a large and unknown collection of *qnr* genes that could be mobilized into pathogens.

**Results:**

In this study we used computational methods to screen genomes and metagenomes for novel *qnr* genes. In contrast to previous studies, we analyzed an almost 20-fold larger dataset comprising almost 13 terabases of sequence data. In total, 362,843 potential *qnr* gene fragments were identified, from which 611 putative *qnr* genes were reconstructed. These gene sequences included all previously described plasmid-mediated *qnr* gene families. Fifty-two of the 611 identified *qnr* genes were reconstructed from metagenomes, and 20 of these were previously undescribed. All of the novel *qnr* genes were assembled from metagenomes associated with aquatic environments. Nine of the novel genes were selected for validation, and six of the tested genes conferred consistently decreased susceptibility to ciprofloxacin when expressed in *Escherichia coli*.

**Conclusions:**

The results presented in this study provide additional evidence for the ubiquitous presence of *qnr* genes in environmental microbial communities, expand the number of known *qnr* gene variants and further elucidate the diversity of this class of resistance genes. This study also strengthens the hypothesis that environmental bacterial communities act as sources of previously uncharacterized qnr genes.

**Electronic supplementary material:**

The online version of this article (doi:10.1186/s12864-017-4064-0) contains supplementary material, which is available to authorized users.

## Background

Fluoroquinolones are widely used synthetic broad-spectrum antibiotics that inhibit the type II topoisomerase complexes essential for bacterial DNA replication. There are three main mechanisms of resistance to fluoroquinolones: 1) mutations in the target enzyme reducing the binding affinity of the antibiotic, 2) efflux pumps facilitating export of the antibiotic, and 3) *qnr* genes encoding proteins thought to sterically prevent the antibiotic to interact with the topoisomerase enzymes [1]. In contrast to fluoroquinolone resistance mechanisms such as chromosomal mutations and efflux pumps, *qnr* genes are often located on mobile plasmids and can be shared between bacterial cells through the process of horizontal gene transfer. The first plasmid-mediated fluoroquinolone resistance gene family, *qnrA*, was discovered in 1998 [2], and since then, five additional families have been described: *qnrS* [3], *qnrB* [4], *qnrC* [5], *qnrD* [6], and most recently *qnrVC* [7]. Novel variants within the established families have been reported over the last years, bringing the current total number of mobile *qnr* gene variants across all families to 115 [8]. In addition, sequence homology searches have revealed *qnr* genes on chromosomes from a wide range of bacterial species [9, 10]. Similarly to many other resistance genes, *qnr* genes are hypothesized to have originated from harmless environmental bacteria and subsequently mobilized into human pathogens [11–13]. Recent reports suggest that *qnr* genes are ubiquitously present in microbial communities from many environments, including marine and lake water, soil, river sediment, and the human microbiome [11, 14–17]. It is therefore possible that these communities also maintain a large collection of novel *qnr* genes that have not yet been transferred to, or discovered in, pathogenic bacteria. Describing this unknown diversity of *qnr* genes is important to fully understand their biological and ecological roles. It also has the potential to uncover currently undescribed resistance genes that might appear in clinical settings in the future [18–20].

Modern high-throughput DNA sequencing technologies have enabled direct analysis of the DNA from bacterial communities without the need for cultivation of individual isolates. The resulting metagenomic data provides information on the combined set of genes maintained by the microorganisms present in the sample [21, 22]. In Boulund et al. (2012) we used a computational method to screen nucleotide sequences from metagenomes for novel *qnr* genes. By analyzing approximately 700 gigabytes of sequence data, evidence of several novel *qnr* genes could be identified. Two of the identified genes selected for experimental validation were confirmed to confer decreased fluoroquinolone susceptibility in an *E. coli* model system [23]. Over the last years, the body of metagenomic data has grown, much thanks to the continuously increasing throughput of shotgun sequencing technologies [24, 25]. In particular, the widely used Illumina sequencing platforms have provided the ability to characterize bacterial communities at an unprecedented sequencing depth [26, 27]. However, the generated metagenomic sequencing data is extensive and highly fragmented, and the identification of novel *qnr* genes therefore requires computationally efficient methods with high sensitivity and specificity.

In this report, we expand upon the work presented in Boulund et al. (2012). Here, we modified and improved the approach and also analyzed a substantially larger data set containing more than 12 terabases of sequence data from bacterial genomes and metagenomes. We identified 362,843 *qnr* gene fragments from which 611 *qnr* gene sequences could be reconstructed. In total 52 *qnr* genes were reconstructed from metagenomic data, and 20 of these were predictions of novel and previously undescribed *qnr* variants. Nine novel *qnr* genes were selected for experimental validation, and six of these (88.8%) conferred consistently reduced susceptibility against fluoroquinolone in *E. coli*.

## Methods

In this study, we applied an extended and improved version of the method for identification of novel resistance genes described in Boulund et al. (2012). The method has been extensively validated on short nucleotide fragments produced by next generation sequencing platforms [10]. In particular, the sensitivity to identify 100 nucleotide long fragments from novel *qnr* genes is 94% with a specificity of 99.8% against other non-qnr pentapeptide repeat proteins. Details for the performance for individual qnr classes are available in Boulund et al. 2012 [10]. The original method operated by translating each nucleotide sequence into amino acids in all six reading frames. Then, a profile hidden Markov model (HMM), based on a multiple alignment of 66 well-characterized plasmid-mediated *qnr* genes, was used to score each fragment based on its sequence [10]. Discrimination between fragments from *qnr* genes and fragments of other origins (including fragments from pentapeptide proteins without fluoroquinolone resistance phenotype) was done by a classifier based on alignment bit score and fragment length. Finally, a clustering step was used to group the *qnr* fragments into clusters representing full-length genes. Complete details are available in Boulund et al. (2012). In this work, we have extended the previous method to make it applicable to larger volumes of data than was feasible before. In particular, the management of the input sequence data has been optimized by minimizing the storage of translated amino acid sequence data and other intermediate files. The last step, where the previous approach clustered all identified putative *qnr* fragments, has also been modified. The reads classified as containing *qnr* fragments are now assembled using metagenome assembly methods, instead of merely clustering them. This makes the method applicable to larger sets of fragmented DNA sequence data, such as those commonly produced in recent metagenomic studies, characterized by short-read high-throughput sequencing techniques (e.g. Illumina sequencing).

In this study, the HMM-based method was used to search for the presence of new *qnr* genes in a large collection of nucleotide sequence data sets, including metagenomes (from human-associated microbial communities, polluted environments, and waste water treatment plants), fully assembled bacterial genomes, and assorted nucleotide sequences from public repositories (e.g. NCBI Genbank). All sequence data (Table 1) were downloaded between May and December 2015 and processed as follows. Sequence files in FASTQ format were quality filtered and converted to FASTA using the FASTX-toolkit version 0.13.2 [28]. Quality filtering was performed with fastq_quality_filter; option: “-q 30”, and FASTQ to FASTA conversion was done with fastq_to_fasta (default settings). All nucleotide data was translated into peptide sequences in all six reading frames using EMBOSS transeq version 6.3.1 [29]; options: “-frame 6 -Table 11”. After translation, HMMER3 (hmmsearch) version 3.1b1 [30] was used together with the hidden Markov model constructed from experimentally verified plasmid-mediated *qnr* genes [10] to search the data sets for the presence of *qnr* fragments; options: “--domtblout -E 1000 --domE 1000”. All fragments matching the HMM were filtered using the fragment length dependent filtering criteria from Boulund et al. (2012). Both read pairs were retrieved for each fragment passing the filtering (even if only one of them matched the HMM), and then used in gene assembly for each individual data set. The paired-end reads were trimmed using Trim Galore! [31] version 0.4.1, using default settings. Assembly of the filtered retrieved paired-end reads was done using the SPAdes assembler version 3.7.0, option: “--meta”. The contigs produced in the assembly were then scored and evaluated against the HMM, using the same length dependent filtering criteria as above, to further validate each assembled sequence.

**Table 1:** Summary of the findings and data sets searched in this study

Data set	Size (nt)	Sequences	Median seq. Length	Number of *qnr* fragments^a^	Assembled *qnr* sequences >200 aa^b^	
Metagenomic					Previously known	Putatively novel
Human microbiomes [43]	3.69 × 10^12^	3.90 × 10^10^	93	83,485 (2.14 × 10^−6^)	1	0
Human intestinal tract [34]	3.71 × 10^11^	5.22 × 10^9^	75	12,966 (2.49 × 10^−6^)	0	0
Human intestinal tract [53]	7.10 × 10^10^	4.41 × 10^8^	148	264 (5.99 × 10^−7^)	1	0
Waste water treatment plant [54]	4.82 × 10^11^	5.18 × 10^9^	101	19,458 (3.76 × 10^−6^)	4	2
Antibiotic-polluted Indian lake [55]	6.75 × 10^9^	6.68 × 10^7^	101	57,415 (8.59 × 10^−4^)	5	0
Antibiotic-polluted Indian soil [35]	4.73 × 10^10^	4.68 × 10^8^	101	840 (1.80 × 10^−6^)	0	0
Antibiotic-polluted Indian river sediment [56]	3.89 × 10^10^	3.85 × 10^8^	101	67,021 (1.74 × 10^−4^)	4	1
Indian well water [35]	7.32 × 10^10^	7.25 × 10^8^	101	1704 (2.35 × 10^−6^)	3	0
Oil spill [57]	2.75 × 10^11^	2.72 × 10^9^	101	12,533 (4.60 × 10^−6^)	0	3
Pune river sediments [58]	3.9 × 10^11^	3.11 × 10^9^	126	4805 (1.55 × 10^−6^)	5	4
Wadden sea [59]	8.4 × 10^9^	5.23 × 10^7^	161	723 (1.38 × 10^−5^)	0	5
Tara oceans [60]	7.20 × 10^12^	7.25 × 10^10^	101	100,795 (1.39 × 10^−6^)	2	5
Other						
NCBI nt	5.22 × 10^10^	2.07 × 10^7^	740	709 (3.42 × 10^−5^)	466	42
NCBI RefSeq bacterial	9.63 × 10^9^	5.24 × 10^3^	1,176,248	54 (1.03 × 10^−2^)	49	2
NCBI env_nt	9.55 × 10^9^	2.07 × 10^7^	117	71 (3.43 × 10^−6^)	3	4
Totals:	1.27 × 10^13^	1.30 × 10^11^	N/A	362,843 (2.79 × 10^−6^)	611	72

^a^Relative abundance given in parenthesis^b^Both putatively novel and previously known sequences are included in this listing

Nine assembled full-length sequences were selected for experimental validation, based on their novelty (no identical sequences in publicly available databases such as NCBI GenBank) and similarity to previously described *qnr* variants (priority given to sequences with high similarity to plasmid-mediated *qnr*). Predicted genes were considered novel based on the criteria presented in Jacoby et al. (2008) [32]. The *qnr* gene candidates were synthesized with KpnI and BamHI restriction sites attached to the 5′ and 3′ ends, respectively. The genes were subcloned into the expression vector pZE21 (EXPRESSYS, Ruelzheim, Germany) under the control of the inducible promoter P_LtetO-1_ by utilizing the attached restriction sites [33]. The generated plasmids were electroporated into *E. coli* C600Z1 (EXPRESSYS). Previously described C600Z1 strains harboring pZE21 plasmids without any gene insert or with either *qnrA1*, *qnrB1,* or *qnrVC1* inserted, were included as controls [23]. The strains carrying the different pZE21 plasmids were cultured overnight on Mueller-Hinton agar (MHA) (Oxoid, Basingstoke, UK) supplemented with kanamycin (50 μg/mL) and subjected to ciprofloxacin susceptibility testing with Etest® gradient strips (BioMérieux SA, Marcy l’Etoile, France) according to the manufacturer’s instructions. The tests were performed on MHA containing the inducer anhydrotetracycline (250 ng/mL) as well as on unsupplemented MHA. *E. coli* ATCC 25922 was used as quality control strain.

## Results

In total, 12.7 terabases of nucleotide data distributed over 130 billion sequences were screened for novel *qnr* genes. The data consisted of metagenomes (12.7 terabases; 99.41%), NCBI RefSeq bacterial genomes (9.63 gigabases; 0.08%) and the NCBI nt database (62 gigabases; 0.41%) (Table 1). In total, 362,843 sequences were classified as fragments from *qnr* genes, which corresponded to an overall relative abundance of 2.79 × 10^−6^. The abundance of *qnr* gene fragments in metagenomes ranged from 5.99 × 10^−7^ to 8.59 × 10^−4^, where the highest levels were found in samples from river and lake sediments contaminated with fluoroquinolones (Table 1). The method identified 611 different *qnr* gene sequences longer than 200 amino acids across all data sets. As expected, most of the sequences (559; 91.5%) were previously characterized *qnr* sequences present in NCBI GenBank and NCBI RefSeq bacterial. Of these, 78 sequences were not previously annotated as *qnr* genes (Additional file 1: Table S1). Of the 5232 analyzed genomes, 49 (0.93%) carried *qnr* genes. In particular, three genes were found in the chromosomes of *Vibrio nigripulchritudo* str SFn1 (NC_022543.1), *Vibrio campbellii* ATCC BAA 1116 (NC_022270.1) and *Serratia liquefaciens* ATC 27592 (NC_021741.1). These species have to the authors’ best knowledge not been previously reported in the literature to carry *qnr* genes. A total of 52 (8.51%) full-length sequences were assembled from metagenomic data, out of which 20 were putatively novel *qnr* genes (either not present or not annotated as a fluoroquinolone resistance gene in Genbank). Full-length *qnr* genes (>200 aa) were assembled from all data sets but two: one with human gut metagenomes [34] and one with soil metagenomes [35]. All predicted *qnr* sequences are presented in (Additional file 2).

**Fig. 1:**
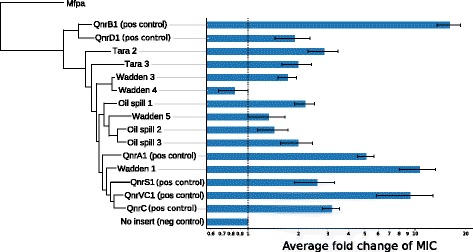
Gene tree showing the relationships between previously known plasmid-mediated qnr sequences and the sequences validated in this work. The gene tree was produced with ETE 3 using the “standard_fasttree” workflow [50–52]. The non-qnr peptapeptide repeat protein MfpA was used as an outlier to root the tree. The bar plot shows fold change of ciprofloxacin minimum inhibitory concentrations for all the tested sequences from three separate measurements each. MIC values for QnrD1, QnrS1, and QnrC for the first time point were taken from our previous study [23]. Wadden 4 did not display an average increase in MIC. Refer to Table 2 for a detailed listing of all sequences discovered in this work, and to Additional file 5: Table S3 for the raw MIC measurement data

The 20 novel *qnr-*like sequences identified in this work had a length between 206 and 223 amino acids and showed, on average, 57.12% amino acid  sequence identity to previously described plasmid-mediated qnr genes (Table 2). Out of the 20 novel *qnr* sequences identified, nine assembled full-length sequences were selected for experimental validation by gene synthesis and transformation into an *E. coli* host. Induced expression of six of the selected sequences resulted in an up to 11-fold increase of the ciprofloxacin minimum inhibitory concentration (MIC) (0.016 to 0.19 mg/L) (Fig. 1). No increase in MIC could be seen for Wadden 4, while Wadden 5 and Oil spill 2 showed inconsistent results between the replicated experiments. Phylogenetic analysis showed that all of the validated genes formed their own clades within the span of the established plasmid-mediated *qnr* families. Sequences Wadden 3 and Wadden 4 formed one small subclade, as did Oil spill 2 and Oil spill 3. All other validated genes formed their own individual clades. A complete tree containing: 1) all sequences discovered in this work, 2) all plasmid-mediated *qnr*, and 3) *mfpa,* is available as an additional figure (Additional file 3: Figure S1).

**Table 2:** Assembled putatively novel qnr sequences discovered in metagenomic datasets

Source data set	Identifier	Assembled sequence length (aa)	Average measured MIC (mg/L)	Most similar plasmid-mediated *qnr*	Identity with most similar pm-*qnr (%)*	Best match in Genbank (% identity)	Comment
Human microbiomes [43]	HMP 1	206	-	QnrB10 and QnrB62	97.04	AFY16910.1(98%)	Possible misassembly: Stop codon in position 30
WWTP [54]	WWTP 1	207	-	QnrB15	61.76	WP_056930987.1(94%)	
WWTP [54]	WWTP 2	220	-	QnrA3	47.93	WP_025325370.1(95%)	10 aa difference to chromosomal variant
WWTP [54]	WWTP 3^a^	220	-	QnrA3	47.93	WP_005328535.1(100%)	Identical to sequence in Genbank
WWTP [54]	WWTP 4^a^	219	-	QnrA3	46.76	WP_041212172.1(100%)	Identical to sequence in Genbank
Antibiotic-polluted Indian lake [55]	Lake 1^a^	220	-	QnrVC4	99.54	WP_000361703.1(100%)	Identical to sequence in Genbank
Antibiotic-polluted Indian river sediment [56]	APRS 1^a^	220	-	QnrVC4	99.54	WP_000361703.1(100%)	Identical to sequence in Genbank; Lake 1
Antibiotic-polluted Indian river sediment [56]	APRS 2	207	-	QnrA3	47.06	WP_041212172.1(99%)	Missing start codon
Indian well water [35]	Well 1^a^	220	-	QnrVC4	99.54	WP_000361703.1(100%)	Identical to sequence in Genbank
Oil spill [57]	Oilspill 1	220	0.037	QnrVC1	65.44	WP_019613380.1 (82%)	
Oil spill [57]	Oilspill 2	220	0.025	QnrVC1	64.98	WP_019613380.1 (80%)	
Oil spill [57]	Oilspill 3	220	0.034	QnrVC1	62.21	WP_057181431.1(77%)	
Pune river sediments [58]	Pune 1	217	-	QnrB27	62.15	WP_056930987.1(95%)	
Pune river sediments [58]	Pune 2	219	-	QnrA3	47.69	WP_005340643.1(99%)	1 aa difference to chromosomal variant
Pune river sediments [58]	Pune 3	220	-	QnrA3	49.77	WP_044798915.1(99%)	3 aa difference to AhQnr
Pune river sediments [58]	Pune 4^a^	219	-	QnrA3	46.76	WP_042864588.1(100%)	Identical to sequence in Genbank
Wadden sea [59]	Wadden 1	220	0.16	QnrVC5	73.73	WP_036748004.1(76%)	
Wadden sea [59]	Wadden 2	220	-	QnrVC6	73.73	WP_007624600.1(75%)	
Wadden sea [59]	Wadden 3	219	0.029	QnrA6	62.96	WP_009385382.1(77%)	
Wadden sea [59]	Wadden 4	219	0.014	QnrVC7	66.67	WP_009385382.1(76%)	
Wadden sea [59]	Wadden 5	220	0.073	QnrA3	57.14	WP_006228785.1(66%)	
Tara oceans [60]	Tara 1^a^	219	-	QnrVC1	60.65	WP_005374358.1(100%)	Identical to sequence in Genbank
Tara oceans [60]	Tara 2	222	0.045	QnrA3	52.29	WP_045976340.1(60%)	
Tara oceans [60]	Tara 3	220	0.032	QnrVC5	53.46	WP_012534242.1(58%)	
Tara oceans [60]	Tara 4	223	-	QnrS2	32.57	BAH90541.1(49%)	
Tara oceans [60]	Tara 5	220	-	QnrA2	32.72	EPJ45142.1(38%)	
Tara oceans [60]	Tara 6	221	-	QnrA3	31.19	EPJ45142.1(37%)	

^a^Assembled sequence identical to already existing sequence in Genbank

## Discussion

In this report, we developed an improved computational approach, appropriate also for deeply sequenced short-read metagenomes, and applied it to screen for new *qnr* genes in collections of DNA sequence data almost totaling 13 terabases. In contrast to what we previously achieved in [10], this study screened an almost 20 times larger dataset, this time dominated by short read metagenomic data produced by next generation DNA sequencing. This also makes this study, to the authors’ best knowledge, the largest and most exhaustive screening of novel *qnr* genes in publicly available nucleotide sequence datasets to date. Our study shows that *qnr*-like genes are widespread in different environments and thus, supports the notion that *qnr* genes could have originated from harmless environmental bacteria. We identified 20 potentially new, full-length gene sequences, all from metagenomic datasets, which have previously not been described or associated with fluoroquinolone resistance. Validation experiments, where the identified putative novel *qnr* genes were expressed in an *E. coli* host, were used to confirm the fluoroquinolone resistance phenotype for six out of nine selected genes, reinforcing confidence in the performance of the predictive model.

The general method applied in this work has previously been shown to reliably predict novel *qnr* genes in sequence data. In particular, extensive cross-validation experiments have demonstrated high true positive rates while maintaining low false positive rates, even for nucleotide fragments as short as 100 bases [10]. Previous predictions based on this method have been experimentally shown to provide increased fluoroquinolone resistance, yielding minimum inhibitory concentration in the same range as other *qnr* genes, when expressed in *E. coli* [23]. Moreover, the method was able to identify all known plasmid-mediated and chromosomally located *qnr* genes in NCBI Genbank and RefSeq, further underlining the validity of the predictions. In addition, three families of *qnr* genes (*qnrD*, *qnrS* and *qnrVC*) were identified and fully assembled from the metagenomes sampled from fluoroquinolone polluted river sediments collected up and downstream from an industrial waste water treatment plant in Patancheru, near Hyderabad, India [15, 36, 37]. These specific gene families have previously been shown to be the most abundant in these samples using other molecular techniques, such as 454 pyrosequencing and quantitative PCR [15, 35]. The accuracy of the method was further emphasized by the fact that six out of nine tested genes consistently conferred the predicted resistance phenotype in *E. coli*. Thus, taken together, our results show that the method is reliable and can correctly identify known and unknown *qnr* genes present in fragmented sequence data. This also implies that the great majority of the genes found in this study that were not validated are also likely to be correctly predicted functional *qnr* genes.

Our results show that *qnr* genes are present in the bacterial chromosomes of 49 (0.93%) of the 5242 analyzed genomes from RefSeq (Additional file 2; Additional file 4: Table S2). This agrees well with reports from previous studies showing that *qnr* genes are carried by a wide range of different bacterial species [9, 10, 14, 38, 39]. Chromosomal and plasmid-mediated *qnr* genes have also been shown to be almost ubiquitously present in e.g. soil, sediment, aquatic, and human microbiomes [9, 10, 14, 38, 39], which is reflected in our analysis, where fragments from potential *qnr* genes were identified in all examined metagenomes. Metagenomes from river and lake sediments polluted by fluoroquinolones had the highest abundance of *qnr* fragments, suggesting that a continuous selection pressure effectively enriches for resistance genes, increasing their relative abundance in the sequenced communities. Interestingly, *qnr* gene abundances were especially low in the metagenomes from the human microbiome (Table 1). This is in line with previous studies that have demonstrated low abundances of the six known plasmid-mediated *qnr* gene families in the human gut microflora [17, 40, 41]. Of the 80,000 potential fragments identified in the Human Microbiome Project (764 samples from 300 individuals), only the previously characterized qnrB62 was fully reconstructed. Instead, the majority of the fragments assembled into contigs that, based on their full length sequences, were not classified as *qnr* and thus disregarded in the analysis. This indicates that a large proportion of the fragments identified in the human microbiome are likely to be false positives and thus not from true *qnr* genes. One possible explanation for the lack of fully reconstructed *qnr* genes from the human microbiome data could be that the abundance of *qnr* genes is very low. This, possibly combined with a high species diversity between individuals [34, 42–44], may negatively influence our ability to completely assemble any *qnr* genes from these data. Our results indicate, however, that no known or novel *qnr* genes are ubiquitously spread in high abundance across the metagenomes of the individuals included in this study, suggesting that horizontally transferrable *qnr* fluoroquinolone resistance genes are rare in the human microbiome. Studies using qPCR, however, indicate that *qnr* genes can be more common in the fecal flora of people from certain regions, e.g. India [35].

Out of the sequences validated in our *E. coli* model, all but three caused an increased MIC of ciprofloxacin in *E. coli* when overexpressed. The strain carrying the sequence Wadden 4 showed no increase in MIC. For Wadden 5 and Oil spill 2, an increase in MIC was only detectable in one and two of the three experiments, respectively. This suggest that these three genes may lack the ability to induce a resistance phenotype to ciprofloxacin. However, the phylogenetic analysis showed that all these three genes were not more distant to the known mobile *qnr* genes than other genes predicted in this study. For example, Wadden 4 formed a small subclade together with Wadden 3, which provided a minor (2-fold) increase in MIC. This may suggest some of these genes were not expressed properly in our assay. However, all other validated sequences exhibited average MIC fold changes between 2 to 10.6, which is in line with representatives of the plasmid-mediated *qnr* families that were also included in the validation for reference [45]. Indeed, several of the novel *qnr* genes identified in this study resulted in an increase of the MIC close, or in some cases, above previously identified mobile *qnr* genes. For example, Wadden 1 had an average fold change in MIC of 10.6 (as high as 15.8 in one experiment), which was larger than QnrA1, QnrC, QnrD1, QnrS1 and QnrVC1. In addition, Tara 2 demonstrated an increase in MIC (2.9) that was higher than QnrA1. Thus, our results suggest that several of the novel *qnr* genes identified in this study may, if transferred into pathogens, become future clinical problems and contribute to the growing number of bacteria resisting antibiotic treatment.

The evolutionary distance between the established plasmid-mediated *qnr* gene families is known to be large and the sequence identity between different variants can be as low as 37.61%, as observed between QnrB24 and QnrS5 [46]. Identification of novel *qnr* gene families therefore requires alignment strategies with high sensitivity and specificity. The computational approach used in this study is based on profile HMMs implemented in HMMER3 [30]. Profile HMMs incorporate sequence variability by describing position-specific amino acid distributions and their short-range dependencies, where the parameters are typically estimated from a multiple alignment of a set of representative sequences. It should, however, be pointed out that *qnr* genes exhibits a wide range of other properties that are not specifically modelled by the profile HMM used in this study. One such example is the recently discovered external surface loops, i.e. short regions of the amino acid residue chain that protrude out of the side of the otherwise regular β-helix structure, observed in the majority of the available mobile *qnr* gene structures [47, 48]. Incorporating information about the fold of the protein requires modelling of long-range dependencies that are hard to accurately capture in an HMM. It might therefore be possible to develop a more sophisticated model that could increase the sensitivity and specificity even further. However, increasing model complexity often comes at the cost of a higher computational burden. The profile HMM implemented in HMMER3 provides both satisfactory sensitivity and specificity in combination with high computational performance, making it well suited for application to large amounts of metagenomic sequencing data.

The amount of nucleotide data screened in this work presents substantial computational challenges. In particular, a large proportion of the data (99.44%) consists of metagenomic DNA sequences that are highly fragmented. A common approach when analyzing this type of data is to assemble each metagenome prior to analysis. However, de novo assembly of large data sets is a daunting task that quickly becomes unfeasible because of the computational and memory complexity, in the worst case, exhibiting quadratic growth with increasing number of fragments [49]. Assembly of all the data included in this study would therefore be highly impractical. Instead, our method essentially applies a data reducing filtering approach where fragments unlikely to represent *qnr* genes (based on similarity to the HMM) are excluded prior to assembly. This provided an almost million-fold decrease of the sequence data (Table 1), which made analysis of these large data sets possible while still maintaining a very high sensitivity, granting us the ability to apply sensitive assembly algorithms to stitch together the identified fragments. Because our method allows studies of all individual metagenomic sequence fragments without prior assembly, it is in a better position to identify fragments from low abundance sequences. Normally, fragments from lowly abundant sequences potentially risk being discarded as assembly chaff when all sequence data is assembled simultaneously. Our approach is therefore well-suited also for screening of future metagenomes with even higher sequencing depth.

## Conclusions

We screened more than 12 terabases of DNA for novel *qnr* fluoroquinolone resistance genes and were able to identify 20 putatively novel *qnr* genes. Of the nine selected for experimental validation, six consistently showed an increased MIC to ciprofloxacin at levels comparable to previously established *qnr* variants when expressed in *E. coli*. Our results thus expand the number of *qnr* gene variants and further elucidates the diversity of this important class of resistance genes. The results also reinforce the hypothesis that environmental bacterial communities act as sources of previously uncharacterized *qnr* genes. Identification of novel antibiotic resistance genes before they are mobilized and transferred to human pathogens enables surveillance at an early stage, facilitating implementation of preventive actions to counter the spread of new variants of multiresistant bacteria in clinical environments.

## Additional files

Additional file 1: Table S1. Annotations for matches in NCBI nt and bacterial genomes. Best hit annotations for sequences matched by the hidden Markov model in NCBI nt and bacterial genome sequences. (XLSX 15 kb)

Additional file 2: Assembled putative *qnr* sequences. Description: Assembled putative *qnr* sequences (DOCX 18 kb)

Additional file 3: Figure S1. Complete gene tree. Description: A gene tree containing all assembled putative *qnr* gene sequences identified in metagenomic data sets, including all established *qnr* gene families. Gene tree created from amino acid sequences with ETE 3 using the “standard_fasttree” workflow. Tree visualized with Dendroscope. Sequences discovered in this work highlighted in bold. (PDF 22 kb)

Additional file 4: Table S2. RefSeq genomes with *qnr* genes. A list of RefSeq genomes where our model detected putative *qnr* genes. (DOCX 12 kb)

Additional file 5: Table S3. MIC measurements. Raw data for Fig. 1. MIC measurements of assembled, putatively novel, qnr gene sequences. Measurements were taken on separate days. (XLSX 53 kb)

## Abbreviations

DNA: Deoxyribonucleic acid
HMM: Hidden Markov model
MHA: Mueller-Hinton agar
MIC: Minimum inhibitory concentration
NCBI: National Center for Biotechnology Information
PCR: Polymerase chain reaction
qPCR: Quantitative polymerase chain reaction

## References

[1] Redgrave LS, Sutton SB, Webber M (2014). A., Piddock LJ V. Fluoroquinolone resistance: mechanisms, impact on bacteria, and role in evolutionary success. Trends Microbiol. Elsevier Ltd.

[2] Martínez-Martínez L, Pascual A, Jacoby GA (1998). Quinolone resistance from a transferable plasmid. Lancet.

[3] Hata M, Suzuki M, Matsumoto M. Cloning of a novel gene for quinolone resistance from a transferable plasmid in Shigella flexneri 2b. Antimicrob agents. 2005; 10.1128/AAC.49.2.801-803.2005 PMC547361 15673773

[4] Jacoby G, Walsh K, Mills D. qnrB, another plasmid-mediated gene for quinolone resistance. Antimicrob. agents. 2006;50. 10.1128/AAC.50.4.1178-1182.2006 PMC1426915 16569827

[5] Wang MM, Guo Q, Xu X, Wang X, Ye X, Wu S (2009). New plasmid-mediated quinolone resistance gene, qnrC, found in a clinical isolate of Proteus Mirabilis. Antimicrob Agents Chemother.

[6] Cavaco LM, Hasman H, Xia S, Aarestrup FM (2009). qnrD, a novel gene conferring transferable quinolone resistance in salmonella enterica serovar Kentucky and Bovismorbificans strains of human origin. Antimicrob. Agents Chemother..

[7] Xia R, Guo X, Zhang Y, Xu H (2010). qnrVC-like gene located in a novel complex class 1 integron harboring the ISCR1 element in an Aeromonas Punctata strain from an aquatic environment in Shandong Province, China. Antimicrob. Agents Chemother.

[8] Jacoby GA. qnr Numbering and Sequence [Internet]. Lahey Clin. 2016 [cited 2016 Oct 6]. p. 1. Available from: http://www.lahey.org/qnrStudies/

[9] Velasco C, Rodríguez-Martínez JM, Briales A, Díaz de Alba P, Calvo J, Pascual A (2010). Smaqnr, a new chromosome-encoded quinolone resistance determinant in Serratia marcescens. J Antimicrob Chemother.

[10] Boulund F, Johnning A, Pereira MB, Larsson DGJ, Kristiansson E (2012). A novel method to discover fluoroquinolone antibiotic resistance (qnr) genes in fragmented nucleotide sequences. BMC Genomics.

[11] Poirel L, Liard A, Rodriguez-Martinez J-M, Nordmann P (2005). Vibrionaceae as a possible source of Qnr-like quinolone resistance determinants. J Antimicrob Chemother.

[12] Allen HK, Donato J, Wang HH, Cloud-Hansen KA, Davies J, Handelsman J (2010). Call of the wild: antibiotic resistance genes in natural environments. Nat. Rev. Microbiol. Nature Publishing Group.

[13] Finley R, Collignon P, Larsson DGJ, A. McEwen S, Li X-Z, Ried-Smith R, et al. The scourge of antibiotic resistance: the important role of the environment. Clin. Infect. …. 2013;1–24. 10.1093/cid/cit355 23723195

[14] Sánchez MB, Hernández A, Rodríguez-Martínez JM, Martínez-Martínez L, Martínez JL (2008). Predictive analysis of transmissible quinolone resistance indicates Stenotrophomonas maltophilia as a potential source of a novel family of Qnr determinants. BMC Microbiol.

[15] Kristiansson E, Fick J, Janzon A, Grabic R, Rutgersson C, Weijdegård B (2011). Pyrosequencing of antibiotic-contaminated river sediments reveals high levels of resistance and gene transfer elements. PLoS One.

[16] Flach C-F, Johnning A, Nilsson I, Smalla K, Kristiansson E, DGJ L (2015). Isolation of novel IncA/C and IncN fluoroquinolone resistance plasmids from an antibiotic-polluted lake. J Antimicrob Chemother.

[17] Bengtsson-Palme J, Angelin M, Huss M, Kjellqvist S, Kristiansson E, Palmgren H, et al. The human gut microbiome as a transporter of antibiotic resistance genes between continents. Antimicrob. Agents Chemother. 2015;AAC.00933-15. 10.1128/AAC.00933-15 PMC4576037 26259788

[18] Perry JA, Wright GD (2013). The antibiotic resistance “mobilome”: searching for the link between environment and clinic. Front Microbiol.

[19] Vaz-Moreira I, Nunes OC, Manaia CM (2014). Bacterial diversity and antibiotic resistance in water habitats: searching the links with the human microbiome. FEMS Microbiol Rev.

[20] Bengtsson-Palme J, Larsson DGJ (2015). Antibiotic resistance genes in the environment: prioritizing risks. Nat. Rev. Microbiol. Nature Publishing Group.

[21] Tringe SG (2005). Von Mering C, Kobayashi a, Salamov a a, Chen K, Chang HW, et al. comparative metagenomics of microbial communities. Science.

[22] Zhu A, Sunagawa S, Mende DR, Bork P (2015). Inter-individual differences in the gene content of human gut bacterial species. Genome Biol.

[23] Flach C-F, Boulund F, Kristiansson E, Larsson DGJ. Functional verification of computationally predicted qnr genes. Ann Clin Microbiol Antimicrob. 2013;12:34. 10.1186/1476-0711-12-34 PMC4222258 24257207

[24] Loman NJ, Constantinidou C, Chan JZM, Halachev M, Sergeant M, Penn CW (2012). High-throughput bacterial genome sequencing: an embarrassment of choice, a world of opportunity. Nat Rev Microbiol Nature Publishing Group.

[25] Cochrane G, Alako B, Amid C, Bower L, Cerdeño-Tárraga A, Cleland I (2013). Facing growth in the European nucleotide archive. Nucleic Acids Res.

[26] Gilbert JA, Dupont CL (2011). Microbial Metagenomics: beyond the genome. Ann Rev Mar Sci Annual Reviews.

[27] Schmieder R, Edwards R (2012). Insights into antibiotic resistance through metagenomic approaches. Future Microbiol.

[28] Gordon A, Hannon GJ. Fastx-toolkit. FASTQ/A short reads pre-processing tools (unpublished) 2010.

[29] Rice P, Longden I, Bleasby A. The European Molecular Biology Open Software Suite EMBOSS : The European Molecular Biology Open Software Suite 2000;16:2–3. 10.1016/s0168-9525(00)02024-2 10827456

[30] Eddy SR (2011). Accelerated profile HMM searches. PLoS Comput Biol.

[31] Krueger F (2015). Trim galore!: a wrapper tool around Cutadapt and FastQC to consistently apply quality and adapter trimming to FastQ files.

[32] Jacoby G, Cattoir V, Hooper D, Martínez-Martínez L, Nordmann P, Pascual A (2008). qnr Gene nomenclature. Antimicrob. Agents Chemother.

[33] Lutz R, Bujard H (1997). Independent and tight regulation of transcriptional units in escherichia coli via the LacR/O, the TetR/O and AraC/I1-I2 regulatory elements. Nucleic Acids Res.

[34] Qin J, Li R, Raes J, Arumugam M, Burgdorf KS, Manichanh C (2010). A human gut microbial gene catalogue established by metagenomic sequencing. Nature.

[35] Rutgersson C, Fick J, Marathe N, Kristiansson E, Janzon A, Angelin M (2014). Fluoroquinolones and qnr genes in sediment, water, soil, and human fecal flora in an environment polluted by manufacturing discharges. Environ Sci Technol.

[36] Larsson DGJ, de Pedro C, Paxeus N (2007). Effluent from drug manufactures contains extremely high levels of pharmaceuticals. J Hazard Mater.

[37] Fick J, Soderstrom H, Lindberg RH, Phan C, Tysklind M, Larsson DGJ (2009). Contamination of surface, ground, and drinking water from pharmaceutical production. Environ Toxicol Chem.

[38] Jacoby GA, Hooper DC (2013). Phylogenetic analysis of chromosomally determined qnr and related proteins. Antimicrob Agents Chemother.

[39] Arsène S, Leclercq R (2007). Role of a qnr-like gene in the intrinsic resistance of Enterococcus faecalis to fluoroquinolones. Antimicrob Agents Chemother.

[40] Forslund K, Sunagawa S, Roat Kultima J, Mende D, Arumugam M, Typas A (2013). Country-specific antibiotic use practices impact the human gut resistome. Genome Res.

[41] Nesme J, Cécillon S, Delmont TO, Monier JM, Vogel TM, Simonet P (2014). Large-scale metagenomic-based study of antibiotic resistance in the environment. Curr Biol.

[42] Arumugam M, Raes J, Pelletier E, Le Paslier D, Yamada T, Mende DR (2011). Enterotypes of the human gut microbiome. Nature.

[43] Huttenhower C, Gevers D, Knight R, Abubucker S, Badger JH, Chinwalla AT (2012). Structure, function and diversity of the healthy human microbiome. Nature Publishing Group.

[44] Penders J, Stobberingh EE, Savelkoul PHM, Wolffs PFG (2013). The human microbiome as a reservoir of antimicrobial resistance. Front Microbiol.

[45] Hooper DC, Jacoby GA. Mechanisms of drug resistance: quinolone resistance. Ann N Y Acad Sci. 2015:1–20. 10.1111/nyas.12830 PMC4626314 26190223

[46] Strahilevitz J, Jacoby GA, Hooper DC, Robicsek A (2009). Plasmid-mediated quinolone resistance: a multifaceted threat. Clin Microbiol Rev.

[47] Xiong X, Bromley EHC, Oelschlaeger P, Woolfson DN, Spencer J (2011). Structural insights into quinolone antibiotic resistance mediated by pentapeptide repeat proteins: conserved surface loops direct the activity of a Qnr protein from a gram-negative bacterium. Nucleic Acids Res.

[48] Vetting MW, Hegde SS, Wang M, Jacoby GA, Hooper DC, Blanchard JS (2011). Structure of QnrB1, a plasmid-mediated fluoroquinolone resistance factor. J Biol Chem.

[49] Miller JR, Koren S, Sutton G (2010). Assembly algorithm for next-Ganeration sequencing data. Genomics.

[50] Sievers F, Wilm A, Dineen D, Gibson TJ, Karplus K, Li W (2011). Fast, scalable generation of high-quality protein multiple sequence alignments using Clustal omega. Mol Syst Biol.

[51] Huerta-Cepas J, Serra F, Bork P (2016). ETE 3: reconstruction, analysis, and visualization of Phylogenomic data. Mol Biol Evol.

[52] Price MN, Dehal PS, Arkin AP. FastTree 2 - approximately maximum-likelihood trees for large alignments. PLoS One. 2010;5 10.1371/journal.pone.0009490 PMC2835736 20224823

[53] Qin J, Li Y, Cai Z, Li S, Zhu J, Zhang F (2012). A metagenome-wide association study of gut microbiota in type 2 diabetes. Nature Nature Publishing Group.

[54] Bengtsson-Palme J, Hammarén R, Pal C, Östman M, Björlenius B, Flach C-F (2016). Elucidating selection processes for antibiotic resistance in sewage treatment plants using metagenomics. Sci Total Environ Elsevier BV.

[55] Bengtsson-Palme J, Boulund F, Fick J, Kristiansson E, Larsson DGJ (2014). Shotgun metagenomics reveals a wide array of antibiotic resistance genes and mobile elements in a polluted lake in India. Front Microbiol.

[56] Pal C, Bengtsson-palme J, Kristiansson E, Larsson DGJ (2016). The structure and diversity of human, animal and environmental resistomes. Microbiome Microbiome.

[57] Mason OU, Hazen TC, Borglin S, Chain PSG, Dubinsky EA, Fortney JL (2012). Metagenome, metatranscriptome and single-cell sequencing reveal microbial response to Deepwater Horizon oil spill. ISME J.

[58] Marathe NP, Pal C, Gaikwad SS, Jonsson V, Kristiansson E, Larsson DGJ. Untreated urban waste contaminates Indian river sediments with resistance genes to last resort antibiotics. Submitted. 2017; 10.1016/j.watres.2017.07.060 28780361

[59] Strous M, Kraft B, Bisdorf R, Tegetmeyer HE (2012). The binning of metagenomic contigs for microbial physiology of mixed cultures. Front Microbiol.

[60] Pesant S, Not F, Picheral M, Kandels-Lewis S, Le Bescot N, Gorsky G (2015). Open science resources for the discovery and analysis of Tara oceans data. Sci data.

