# Supplementary material for: Computational discovery and functional validation of novel fluoroquinolone resistance genes in public metagenomic data sets
Source: BMC Genomics. 2017 Sep 2;18:682. doi: 10.1186/s12864-017-4064-0 (PMC5581476; doi:10.1186/s12864-017-4064-0)
Supplement: Supplementary file 2 — Assembled putative qnr sequences. Description: Assembled putative qnr sequences (DOCX 18 kb) [file 12864_2017_4064_MOESM2_ESM.docx]

>HMP_1 NODE_60_length_635_cov_2.53448_ID_19982_5/10-212

GIDMTLALVGEKIDRNRFTGEKVENSTFFN*DLSGADLSGTEFIGCQFYDRESQKGCNFS

RAMLKDAIFKSCDLSMADFRNASALGIEIRHCRAQGADFRGASFMNMITTRTWFCSAYIT

NTNLSYANFSKVVLEKCELWENRWMGAQVLGATFSGSDLSGGEFSTFDWRAANFTHCDLT

NSELGDLDLRGVDLQGVKLDNYX

>Oilspill_1 NODE_3_length_774_cov_2.41586_ID_3426_3/13-229

MMSSNLTYREHDFSQQNLQDTIFENCNFYQCDFSRTDLRDAKFINCTFIETGVSIGCQFN

YADLRDASFKQCKLGMANFQGANGFGAEFRECDLQGANFRKTSFVNHISHQSYFCSVYIT

GCKLSYTNFERQCIEKCDLFENRWTGANLQQASFKGSDLSRCEFSEDSWSQFDIQYCDLT

NSELYGLDPRRVNLNGVKICDWQQEQLLEPLGLIVLP

>Oilspill_2 NODE_2_length_787_cov_13.4399_ID_3424_6/14-230

MPTIKKNYINHDFSGQDLKEMHFEQCNFYECNFKKADLADSKFIECRFIEQGSINGCRFD

YANLPDASFKQCQLAMSDFTGANCLGIEFRSCDLKGANFAKASFENQVSRQVYFCSAYIT

GCNLSYANFAGTRIEKCDLFENRWNGVNLQGASLKHSDLSRSEFSEDVWEQFQIQGCNLT

HTELTGLNIRRVKLDGVMICNWQQEQLLEDLGVIVLP

>Oilspill_3 NODE_1_length_808_cov_16.0983_ID_3422_2/18-234

MPTVNKTFRKHDFSKENLSTMNFENCKFYECNFNRSDLTDAKFIECRFIEQGGINGSTFD

YANLRDASFKHCQLSMASFIGANCLGAEFRSCDLKGADFAKTRFENQISRLSYFCSVYIT

SCNLSYANFASARIEKCDLFENKWIGANLEGASLKGSDLSRSVFSEDAWEQFQLQGCDLT

HTELNGLNIRRVKLDGVKICNWQQEQLLENLGLLILP

>Lake_QnrS2 NODE_2_length_1517_cov_48.788_ID_1714_3/188-404

METYRHTYRHHSFSHQDLSDITFTACTFIRCDFRRANLRDATFINCKFIEQGDIEGCHFD

VADLRDASFQQCQLAMANFSNANCYGIELRECDLKGANFSRANFANQVSNRMYFCSAFIT

GCNLSYANMERVCLEKCELFENRWIGTHLAGASLKESDLSRGVFSEDVWGQFSLQGANLC

HAELDGLDPRKVDTSGIKIASWQQEQLLEALGIVVFP

>Lake_QnrD1_1 NODE_1_length_4376_cov_1703_ID_1712_6/1121-1337

VACMEKHFINEKFSRDQFTGNRVKNIAFSNCDFSGVDLTDTEFVDCSFYDRNSLEGCDFN

RAKLKNASFKSCDLSMSNFKNISALGLEISECLAQGADFRGANFMNMITTRSWFCSAYIT

KTNLSYANFSRVILEKCELWENRWNGTVITGAVFRGSDLSCGEFSSFDWSLADFTGCDLT

GGALGELDARRINLDGVKLDGEQALQLVESLGVIVHR

>Lake_QnrD1_2 NODE_1_length_4376_cov_1703_ID_1712_4/228-444

VACMEKHFINEKFSRDQFTGNRVKNIAFSNCDFSGVDLTDTEFVDCSFYDRNSLEGCDFN

RAKLKNASFKSCDLSMSNFKNISALGLEISECLAQGADFRGANFMNMITTRSWFCSAYIT

KTNLSYANFSRVILEKCELWENRWNGTVITGAVFRGSDLSCGEFSSFDWSLADFTGCDLT

GGALGELDARRINLDGVKLDGEQALQLVESLGVIVHR

>Lake_1 NODE_3_length_1113_cov_18.0652_ID_1716_5/88-304

MDKTDQLYVQADFSHQDMSGQYFKNCKFFCCSFKRANLRDTQFVDCSFIERGELEGCDFS

YSDLRDASFKNCSLSMSYFKGANCFGIEFRECDLKGANFAQASFMNQVSNRMYFCSAYIT

GCNLSYANFERQCIEKCDLFENRWIGANLSGASFKESDLSRGVFSEGCWSQCRLQGCDLS

HSDLYGLDPRKVDLTGVKICSWQQEQLLEQLGLIVVP

>Lake_QnrVC1 NODE_4_length_948_cov_27.1545_ID_1718_5/35-251

MEKSKQLYNQVNFSHQDLQEHIFSNCTFIHCNFKRSNLRDTQFINCTFIEQGALEGCDFS

YADLRDASFKNCQLSMSHFKGANCFGIELRDCDLKGANFSQVSFVNQVSNKMYFCSAYIT

GCNLSYANFEQQLIEKCDLFENRWIGANLRGASFKESDLSRGVFSEDCWEQFRVQGCDLS

HSELYGLDPRKIDLTGVKICSWQQEQLLEQLGVIIVP

>APRS_QnrS1 NODE_2_length_1872_cov_197.506_ID_1662_3/213-429

METYRHTYRHHSFSHQDLSDITFTACTFIRCDFRRANLRDATFINCKFIEQGDIEGCHFD

VADLRDASFQQCQLAMANFSNANCYGIELRECDLKGANFSRANFANQVSNRMYFCSAFIT

GCNLSYANMERVCLEKCELFENRWIGTHLAGASLKESDLSRGVFSEDVWGQFSLQGANLC

HAELDGLDPRKVDTSGIKIASWQQEQLLEALGIVVFP

>APRS_QnrD1 NODE_1_length_2737_cov_650.94_ID_1660_6/1-216

ACMEKHFINEKFSRDQFTGNRVKNIAFSNCDFSGVDLTDTEFVDCSFYDRNSLEGCDFNR

AKLKNASFKSCDLSMSNFKNISALGLEISECLAQGADFRGANFMNMITTRSWFCSAYITK

TNLSYANFSRVILEKCELWENRWNGTVITGAVFRGSDLSCGEFSSFDWSLADFTGCDLTG

GALGELDARRINLDGVKLDGEQALQLVESLGVIVHR

>APRS_1 NODE_4_length_1592_cov_783.282_ID_1668_6/73-289

MDKTDQLYVQADFSHQDMSGQYFKNCKFFCCSFKRANLRDTQFVDCSFIERGELEGCDFS

YSDLRDASFKNCSLSMSYFKGANCFGIEFRECDLKGANFAQASFMNQVSNRMYFCSAYIT

GCNLSYANFERQCIEKCDLFENRWIGANLSGASFKESDLSRGVFSEGCWSQCRLQGCDLS

HSDLYGLDPRKVDLTGVKICSWQQEQLLEQLGLIVVP

>APRS_QnrVC1 NODE_3_length_1755_cov_481.88_ID_1666_2/73-289

MEKSKQLYNQVNFSHQDLQEHIFSNCTFIHCNFKRSNLRDTQFINCTFIEQGALEGCDFS

YADLRDASFKNCQLSMSHFKGANCFGIELRDCDLKGANFSQVSFVNQVSNKMYFCSAYIT

GCNLSYANFEQQLIEKCDLFENRWIGANLRGASFKESDLSRGVFSEDCWEQFRVQGCDLS

HSELYGLDPRKIDLTGVKICSWQQEQLLEQLGVIIVP

>APRS_2 NODE_8_length_610_cov_1.57477_ID_1676_1/1-204

HFEQREVEGEQFSGCRFIGCHFSWLDLGESRFVDCSFYDRERELGCLFQGCDLREASFLR

CDLTLADFGRSQCLGLEMRDCQAMGINFAHASFANRITAKSYFCEAHLTGNNFSYANFES

CLLERCELAGNRWQGANLLGASLAGSDLSGSEFGQIDWTSFNLQGCDLRQCDLSGLDLRR

VNLAGVQINEDQQQGLLEQIGLVX

>Well_QnrD1 NODE_1_length_2013_cov_19.6828_ID_532_1/266-482

VACMEKHFINEKFSRDQFTGNRVKNIAFSNCDFSGVDLTDTEFVDCSFYDRNSLEGCDFN

RAKLKNASFKSCDLSMSNFKNISALGLEISECLAQGADFRGANFMNMITTRSWFCSAYIT

KTNLSYANFSRVILEKCELWENRWNGTVITGAVFRGSDLSCGEFSSFDWSLADFTGCDLT

GGALGELDARRINLDGVKLDGEQALQLVESLGVIVHR

>Well_1 NODE_4_length_1022_cov_3.17063_ID_540_3/72-288

MDKTDQLYVQADFSHQDMSGQYFKNCKFFCCSFKRANLRDTQFVDCSFIERGELEGCDFS

YSDLRDASFKNCSLSMSYFKGANCFGIEFRECDLKGANFAQASFMNQVSNRMYFCSAYIT

GCNLSYANFERQCIEKCDLFENRWIGANLSGASFKESDLSRGVFSEGCWSQCRLQGCDLS

HSDLYGLDPRKVDLTGVKICSWQQEQLLEQLGLIVVP

>Well_QnrVC1 NODE_3_length_1151_cov_5.94799_ID_544_6/97-313

MEKSKQLYNQVNFSHQDLQEHIFSNCTFIHCNFKRSNLRDTQFINCTFIEQGALEGCDFS

YADLRDASFKNCQLSMSHFKGANCFGIELRDCDLKGANFSQVSFVNQVSNKMYFCSAYIT

GCNLSYANFEQQLIEKCDLFENRWIGANLRGASFKESDLSRGVFSEDCWEQFRVQGCDLS

HSELYGLDPRKIDLTGVKICSWQQEQLLEQLGVIIVP

>Pune_QnrB1 NODE_7_length_921_cov_12.9723_ID_3489_5/31-247

GTNMALALVGEKIDRNRFTGEKIENSTFFNCDFSGADLSGTEFIGCQFYDRESQKGCNFS

RAMLKDAIFKSCDLSMADFRNSSALGIEIRHCRAQGADFRGASFMNMITTRTWFCSAYIT

NTNLSYANFSKVVLEKCELWENRWIGAQVLGATFSGSDLSGGEFSTFDWRAANFTHCDLT

NSELGDLDIRGVDLQGVKLDNYQASLLMERLGIAVIG

>Pune_QnrS2 NODE_4_length_1090_cov_3.82995_ID_3483_1/36-252

METYRHTYRHHSFSHQDLSDITFTACTFIRCDFRRANLRDATFINCKFIEQGDIEGCHFD

VADLRDASFQQCQLAMANFSNANCYGIELRECDLKGANFSRANFANQVSNRMYFCSAFIT

GCNLSYANMERVCLEKCELFENRWIGTHLAGASLKESDLSRGVFSEDVWGQFSLQGANLC

HAELDGLDPRKVDTSGIKIASWQQEQLLEALGIVVFP

>Pune_QnrS1 NODE_1_length_1127_cov_22.7211_ID_3477_1/74-290

METYNHTYRHHNFSHKDLSDLTFTACTFIRSDFRRANLRDTTFVNCKFIEQGDIEGCHFD

VADLRDASFQQCQLAMANFSNANCYGIEFRACDLKGANFSRTNFAHQVSNRMYFCSAFIS

GCNLSYANMERVCLEKCELFENRWIGTNLAGASLKESDLSRGVFSEDVWGQFSLQGANLC

HAELDGLDPRKVDTSGIKIAAWQQELILEALGIVVYP

>Pune_QnrVC4 NODE_6_length_952_cov_11.0702_ID_3487_2/17-233

MDKTDQLYVQADFSHQDMSGQYFKNCKFFCCSFKRANLRDTQFVDCSFIERGELEGCDFS

YSDLRDASFKNCSLSMSYFKGANCFGIEFRECDLKGANFAQASFMNQVSNRMYFCSAYIT

GCNLSYANFERQCIEKCDLFENRWIGANLSGASFKESDLSRGVFSEGCWSQCRLQGCDLS

HSELYGLDPRKVDLTGVKICSWQQEQLLEQLGLIVVP

>Pune_QnrVC1 NODE_2_length_1122_cov_18.5108_ID_3479_5/97-313

MEKSKQLYNQVNFSHQDLQEHIFSNCTFIHCNFKRSNLRDTQFINCTFIEQGALEGCDFS

YADLRDASFKNCQLSMSHFKGANCFGIELRDCDLKGANFSQVSFVNQVSNKMYFCSAYIT

GCNLSYANFEQQLIEKCDLFENRWIGANLRGASFKESDLSRGVFSEDCWEQFRVQGCDLS

HSELYGLDPRKIDLTGVKICSWQQEQLLEQLGVIIVP

>Pune_1 NODE_15_length_700_cov_1.90698_ID_3505_4/20-233

VAAPAIVSNERIGRERFTGATVANTHFRNCDFSGADLTGTAFVNCVFYDADTQAGCRFNG

AQLKEARFHQCDLSMCGFAFAKALGLEIVECRAQGADFSNASFMNQITARSWFCSAVIRQ

SNLAYADFSGVTLEKCALPDNRWTGANVAGASFSGSDLSGGDFSALDWRSADFTHCDLTA

SELGDLDLRVVDLEGARLDTLQVAQLMLQLGITV

>Pune_2 NODE_8_length_921_cov_7.12587_ID_3491_2/39-254

MIRGKQFVDVRFEQQDVEGEQFSECRFIGCNFSWLDLGDSRFIDCSFYDRESERGCLLQG

CDLREASFLRCDLTMADCSRSQCLGLEMRDCQAVGINFSHASFANQITAKSYFCEAHLTG

NNFSYASFEGCLLEKCELTGNRWQGANLFGASLAGSDLSGSEFGQIDWTSFKLQGCDLRQ

CDLPGLDLRRVDLQGVQINEDQQQTLLEQIGLVVFP

>Pune_3 NODE_9_length_917_cov_5.44084_ID_3493_5/38-254

GMIKDKCFEGVRFEQQDLEGEQFQGCRFIGCNFSWLDLAECRFVDCSFYDRESEQSCLLQ

GCDLREASFLRCDLTMADCSQSQCLGLELRDCQALGINFSRASFANQITVKSYFCEAHLT

GNNFSYANFEGCLLEQCELSGNRWQGANLFGASLAGSDLSGSEFGQIDWASVNLQGCDLR

QCDLPGLDLRRVNLDGVQINEEQQRALLEQIGLIVFP

>Pune_4 NODE_10_length_908_cov_4.1442_ID_3495_1/8-223

MIKGRCFEGIHFEQQEVEGEQFQGCRFIGCHFSWLDLGESRFVDCSFYDRERELGCLFQG

CDLREASFLRCDLTLADFGRSQCLGLEMRDCQAMGINFAHASFANRITAKSYFCEAHLTG

NNFSYANFESCLLERCELAGNRWQGANLLGASLAGSDLSGSEFGQIDWTSFNLQGCDLRQ

CDLPGLDLRRVNLAGVQINEDQQQGLLEQIGLVVFP

>Qin2012_QnrS1 NODE_1_length_1131_cov_5.36989_ID_70_2/82-298

METYNHTYRHHNFSHKDLSDLTFTACTFIRSDFRRANLRDTTFVNCKFIEQGDIEGCHFD

VADLRDASFQQCQLAMANFSNANCYGIEFRACDLKGANFSRTNFAHQVSNRMYFCSAFIS

GCNLSYANMERVCLEKCELFENRWIGTNLAGASLKESDLSRGVFSEDVWGQFSLQGANLC

HAELDGLDPRKVDTSGIKIAAWQQELILEALGIVVYP

>Tara_QnrS2 NODE_2_length_826_cov_17.7769_ID_236907_5/21-237

METYRHTYRHHSFSHQDLSDITFTACTFIRCDFRRANLRDATFINCKFIEQGDIEGCHFD

VADLRDASFQQCQLAMANFSNANCYGIELRECDLKGANFSRANFANQVSNRMYFCSAFIT

GCNLSYANMERVCLEKCELFENRWIGTHLAGASLKESDLSRGVFSEDVWGQFSLQGANLC

HAELDGLDPRKVDTSGIKIASWQQEQLLEALGIVVFP

>Tara_1 NODE_7_length_761_cov_2.59773_ID_236917_1/5-220

MLKTDLIFERENFSHHDFQNATFKNCHFYMCCFDHADLRDAKFIDCRFIESKALEGCSFR

FANLKDASFTNCMLAMSLFNGANCMGLELRKCDLKGANFQGANFANRVSNTMFFCSAFIT

GCNLTYCNFERVLLEKCDLFENRWNGANLAGATLKGSDLSRCEFSPEQWGTFNVEQCDLT

HVELDGLDVRRVSLFGVKICDWQQEQLLAPFGLIIL

>Tara_2 NODE_5_length_787_cov_11.8962_ID_236913_4/18-236

RNSTMHYQGEQFEGKHFDEDLEAVKFENCRFIDCDFTRAKLSGAEFSGCAFTTSDGDKGC

SFSFADIRDTSFRNCRLALASFRGADGFGAEFRDCDLKGADFRQASFANFITTKSYFCSV

FITGCNLSYADFEGQLFEKCELTENIWRGANLSGVSMDGADLSRGDFSSDSWGTFSLKNC

DLRHVDLHGLDIRRMDLNGVKICDWQQESLLSPLGLVVS

>Tara_3 NODE_3_length_822_cov_35.8188_ID_236909_5/22-238

YTMKNEIFERVDFSGQDLSGQYYSHCQFLGCRFDRCDLTDAIFEHCSFVEPGAQGACSFS

FSKLADARFEHCKMAMVNFKGADCLGIVLNECDLKGADFSQASFSNQINHNSYFCSAVIT

NCNLSYANLSRARLESCKLSGNRWTDANLSGATLQGADLSRGEFSPESWYQCQLQNADLR

HCDLVGLDIRRVDVTGIKITQWQQHELLAQLGIEVEA

>Tara_4 NODE_1_length_845_cov_10.2063_ID_236905_4/31-250

GSRFADEDFSNTNFHDADLAGAVFVNCRFPRCGFRAANLEGTHFERCVFFHPDDGDDGGV

QFDFAILREASFEHCDLTTASFNHVSAYDLTLSHCQLQGADFGEADFRLPIRSNKDLATF

TMEHCNFSYGNLSNTVLTECTLTNNRMIEMTAHNTRLDAANLSGSDISNLHGHGLSLCGA

DLRGAVFNNLDPREIDLRGVHITPDQALLLVEILGAIVDV

>Tara_5 NODE_8_length_738_cov_5.83309_ID_236919_5/2-218

IQDLNFDHCIFEKNEFGDLESLQFTDCIFLECSFNSSSIKETLFENTRFYDADTEKGCTF

QFAQFDGSTFKGCDLTLANFSRACLYRVTMNQCQATGIDFSHATSSHQVGNTVVLSDAWI

NDCNCAYANMKGANFCECDLSDNRFSHSLLDNAVLDNTLLNGCDFHGVESEGLSIKGADL

RDAQIGGLDVRRMDMTGVRINDYQQRILLEAIGMIVD

>Tara_6 NODE_6_length_762_cov_18.0226_ID_236915_5/8-225

GTTFSNVTFEDVTLRGCELEALEFRQCTFLRCRFDSSAFRESTFARCVFFDKDSESGCSL

RFTNLRLARFEDCDLSMCRLDRARLHQIYIARSQGQGLSAREATAVHEIVGGFELAEGSL

VDCNLAYADFTGADLTRCELSGNRLSHAVFDRAILREAQLTDCELHGVSAEGVEIRGADL

RGSELSGLDVREVDLDGVRITVSQQSALLEPLGLIVTD

>Wadden_1 NODE_1_length_1034_cov_14.5026_ID_420_6/67-283

MQSEREVFHQHDFSNQDLSNALFKHCKFHRCDFARANLREAQFEDCSFIEQGEIEGCHFD

YADLRDASFKDCQISMANFKGANCFGIEFRLCDLKGANFSTALFTNQISYKMYFCSAYIT

GCNLSYTNFEKQCMEKCELFENKWVGANLQGASFKQSDLSRSVFSKECWYQFSMQGCDLS

HSELDGLDPRKVDLTGVKICSWQQEQLLEQLGIIVMP

>Wadden_2 NODE_3_length_953_cov_7.82071_ID_424_1/41-257

MKKTNQLYDDTDFTKQDLSDSLFEKCTFIRCDFNHANLRDAHFVDCTFIRQGDIEGCDFG

FCDLRDASFKDCQLSMSNFSGANCFGIELRDCDLKGANFFRASFVNQVSYKMFFCSAYIT

GCNLSYANFERQCIEKCDLFENRWISTNLQGASLKESDLSRGSFSGDIWGQFRMEGCNLS

HSELDGLDPRKVDLTGVKICEWQQEQLLEKLGLIVLP

>Wadden_3 NODE_2_length_1012_cov_5.97283_ID_422_2/55-270

MIYEDKIFNEHSFASQDLSYAKFVKCKFFNCDFSRAQLVETQFEQCSFIEPGAIEGCHFN

YANLQEASFKSCQLSMSQFTGCNCFGIEFRQCDLKGANFSQARFANQISHNVYFCSAYIT

GCNLSYANMERLCIEKCDLFENRWIETNLQGSSLQGSDLSNGEFSSDIWGEFRIRECDLT

DCDLTGLNPTRVDMTGVKIASWQQSQLLEQLGVIVI

>Wadden_4 NODE_4_length_939_cov_6.85294_ID_426_5/46-261

MNYEDKVFNEESFANQDLSYAKFVKCKFFNCDFSRAQLVETQFDQCSFIEQGELEGCDFH

YANLQEASFKNCQLSMSQFVGSNCFGIEFRQCDLKGANFSQARFANQISHNVYFCSAYIT

GCNLSYANLERQCIEKCDLFENRWIDTNLQGTSLQGSDLSNGEFSANIWGDFRIRECDLT

DCDLTGLNPIKVDLTGVKIASWQQLQLLEQLGVIVI

>Wadden_5 NODE_5_length_778_cov_2.60304_ID_428_6/7-223

MNVSGSTFTEEDFSNTDLAGSVFNQCNFYRCSFVRADLTDAKFTNCRFIEPGDIEGCRFE

YAQLKDASFNDCDLSMALFTGAHCLGIEFRSCNMKGCNFSRANFCNYITHNTYFSSAYIT

GCNLSYSNFENQRLEKCDLFENRWNGANLMNASLKGSDLSRCEFSEEQWGQFVIEECNLC

HVDLTGLDLRKVKLKGVMICDWQQEQLLEPFGLCILP

>WWTP_QnrS2 NODE_2_length_1340_cov_32.8638_ID_9213_3/109-325

METYRHTYRHHSFSHQDLSDITFTACTFIRCDFRRANLRDATFINCKFIEQGDIEGCHFD

VADLRDASFQQCQLAMANFSNANCYGIELRECDLKGANFSRANFANQVSNRMYFCSAFIT

GCNLSYANMERVCLEKCELFENRWIGTHLAGASLKESDLSRGVFSEDVWGQFSLQGANLC

HAELDGLDPRKVDTSGIKIASWQQEQLLEALGIVVFP

>WWTP_QnrD1 NODE_1_length_1369_cov_31.5183_ID_9211_6/119-335

VACMEKHFINEKFSRDQFTGNRVKNIAFSNCDFSGVDLTDTEFVDCSFYDRNSLEGCDFN

RAKLKNASFKSCDLSMSNFKNISALGLEISECLAQGADFRGANFMNMITTRSWFCSAYIT

KTNLSYANFSRVILEKCELWENRWNGTVITGAVFRGSDLSCGEFSSFDWSLADFTGCDLT

GGALGELDARRINLDGVKLDGEQALQLVESLGVIVHR

>WWTP_1 NODE_23_length_730_cov_3.80148_ID_9271_4/40-243

AATAATVVSNARIDRDRFTGATVASTHFRNCDFSGADLTGTTFVNCVFYDADTQAGCRFN

GAQLKEARFRQCDLSLCGFAFAKALGLEIVECRAQGADFSRASFMNQITARSWFCSAVIR

QSNLAYADFSGVTLEKCELPDNRWTGASVAGASFSGSDMSGGDFSSLDWRSANFTHCDLS

GSELGDLDLRVVDLEGVRLDALQV

>WWTP_2 NODE_8_length_996_cov_24.0882_ID_9229_3/68-284

GMIKGKCFEGKRFEQQEVEGEQFSECRFIDCNFSWLDLSDSRFVDCSFYDRESERGCLLQ

GCDLREASFLRCDLTLADCSRSQCLGLEMRDCQAVGINFGHASFANQITAKSYFCEAHLT

GNNFSYANFDGCLLEKCELGGNRWQGASLLGASLAGSDLSGSEFGQIDWTSFNLQGCDLR

QCDLPGLDLRRVNLDGVQINEEQQQTLLEQIGLVVFP

>WWTP_3 NODE_27_length_696_cov_29.4446_ID_9287_5/12-228

GMIKGKCFERKRFEQQEVEGQSFSECRFVGCNFSWLDLAECRFVDCSFYERERELGCQLQ

GCDLREASFLRCDLTLADFSRSQCLGLEMRDCQAMGINFAHASFANQITVKSYFCEAHLT

GNNFSYANFDGCLLERCELGGNRWQGASLLGASLAGSDLSGSEFGQIDWTSFNLQGCDLR

QCDLPGLDLRRVNLDGVQINEEQQQGLLEQIGLVVFP

>WWTP_4 NODE_21_length_771_cov_11.3338_ID_9263_3/38-253

MIKGRCFEGVHFEQQEVEGEQFQGCRFIGCHFSWLDLGESRFVDCSFYDRERELGCLFQG

CDLREASFLRCDLTLADFGRSQCLGLEMRDCQAMGINFAHASFANRITAKSYFCEAHLTG

NNFSYANFESCLLERCELAGNRWQGANLLGASLAGSDLSGSEFGQIDWTSFNLQGCDLRQ

CDLPGLDLRRVNLAGVQINEDQQQGLLEQIGLVVFP
