# Supplementary figures and images for: Computational discovery and functional validation of novel fluoroquinolone resistance genes in public metagenomic data sets
Source: BMC Genomics. 2017 Sep 2;18:682. doi: 10.1186/s12864-017-4064-0 (PMC5581476; doi:10.1186/s12864-017-4064-0)

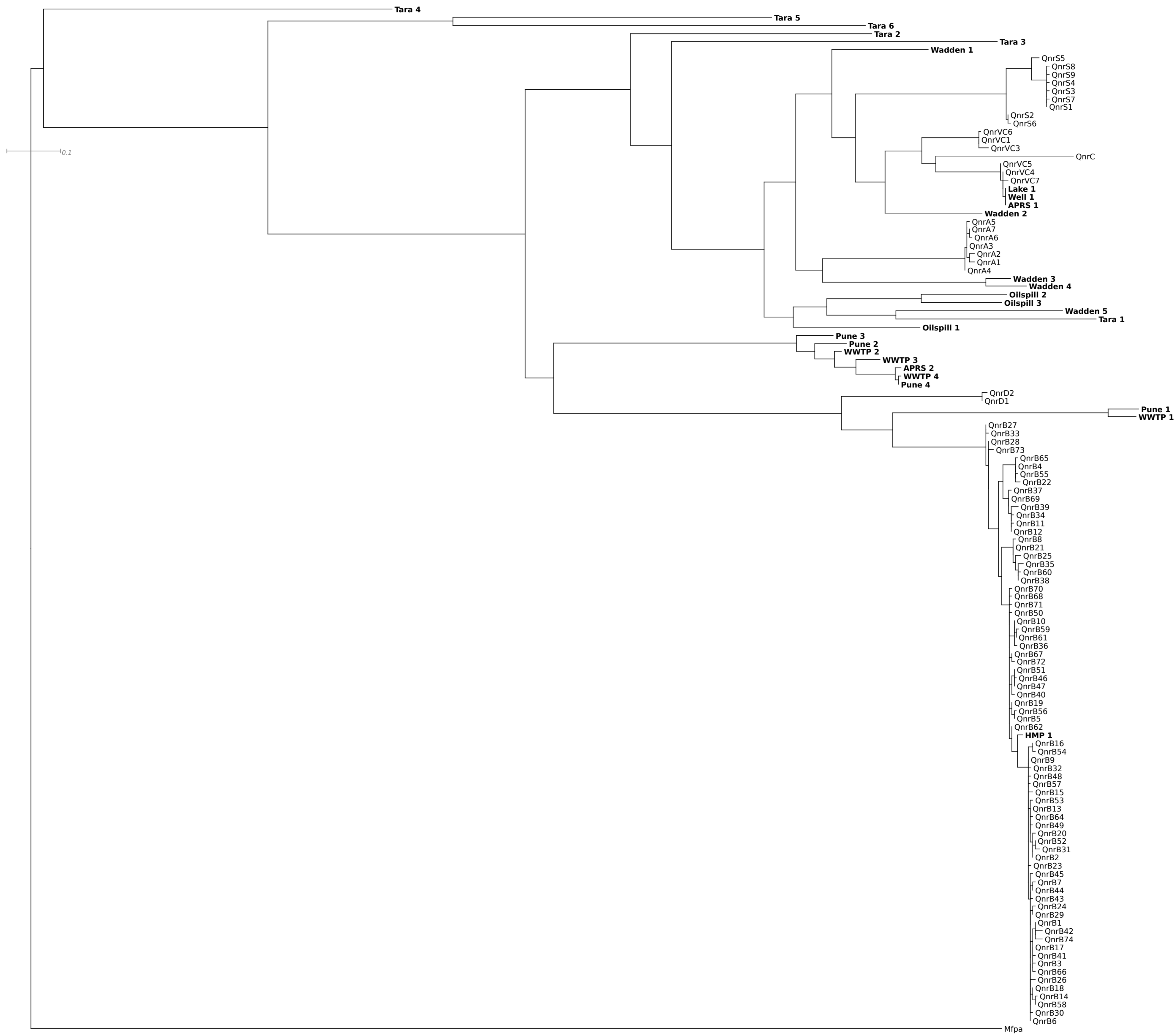

Supplement: Supplementary file 3 — Complete gene tree. Description: A gene tree containing all assembled putative qnr gene sequences identified in metagenomic data sets, including all established qnr gene families. Gene tree created from amino acid sequences with ETE 3 using the “standard_fasttree” workflow. Tree visualized with Dendroscope. Sequences discovered in this work highlighted in bold. (PDF 22 kb) [file 12864_2017_4064_MOESM3_ESM.pdf]
