# Supplementary material for: Computational discovery and functional validation of novel fluoroquinolone resistance genes in public metagenomic data sets
Source: BMC Genomics. 2017 Sep 2;18:682. doi: 10.1186/s12864-017-4064-0 (PMC5581476; doi:10.1186/s12864-017-4064-0)
Supplement: Supplementary file 4 — RefSeq genomes with qnr genes. A list of RefSeq genomes where our model detected putative qnr genes. (DOCX 12 kb) [file 12864_2017_4064_MOESM4_ESM.docx]

# Supplementary table 2: RefSeq genomes with *qnr* genes

RefSeq genome sequences that our model identified *qnr* sequences in. Sequences in **bold** have to the authors’ best knowledge not been mentioned in published material before. Also note that three *Shewanella* genome sequences (*italicized*) appear to contain more than one *qnr* sequence.

NC_021290.1 Aeromonas_hydrophila_ML09-119,_complete_genome
NC_008570.1 Aeromonas_hydrophila_subsp._hydrophila_ATCC_7966_chromosome,_complete_genome
NC_009348.1 Aeromonas_salmonicida_subsp._salmonicida_A449,_complete_genome
NC_015424.1 Aeromonas_veronii_B565_chromosome,_complete_genome
NC_014500.1 Dickeya_dadantii_3937_chromosome,_complete_genome
NC_018107.1 Klebsiella_oxytoca_E718_plasmid_pKOX_R1,_complete_sequence
NC_022224.1 Listonella_anguillarum_M3_chromosome_2,_complete_sequence
NC_006371.1 Photobacterium_profundum_SS9_chromosome_2,_complete_sequence
NC_016818.1 Rahnella_aquatilis_CIP_78.65_=_ATCC_33071_chromosome,_complete_genome
NC_017047.1 Rahnella_aquatilis_HX2_chromosome,_complete_genome
NC_015061.1 Rahnella_sp._Y9602_chromosome,_complete_genome
**NC_021741.1 Serratia_liquefaciens_ATCC_27592,_complete_genome**
NC_020211.1 Serratia_marcescens_WW4,_complete_genome
NC_021591.1 Serratia_plymuthica_4Rx13,_complete_genome
NC_015567.1 Serratia_plymuthica_AS9_chromosome,_complete_genome
NC_021659.1 Serratia_plymuthica_S13,_complete_genome
NC_009832.1 Serratia_proteamaculans_568_chromosome,_complete_genome
NC_015566.1 Serratia_sp._AS12_chromosome,_complete_genome
NC_017573.1 Serratia_sp._AS13_chromosome,_complete_genome
NC_022268.1 Serratia_sp._ATCC_39006_genome
NC_008345.1 Shewanella_frigidimarina_NCIMB_400_chromosome,_complete_genome
NC_010334.1 Shewanella_halifaxensis_HAW-EB4_chromosome,_complete_genome
NC_009092.1 Shewanella_loihica_PV-4_chromosome,_complete_genome
*NC_009901.1 Shewanella_pealeana_ATCC_700345_chromosome,_complete_genome*
NC_011566.1 Shewanella_piezotolerans_WP3_chromosome,_complete_genome
*NC_009831.1 Shewanella_sediminis_HAW-EB3_chromosome,_complete_genome*
*NC_014012.1 Shewanella_violacea_DSS12_chromosome,_complete_genome*
NC_010506.1 Shewanella_woodyi_ATCC_51908_chromosome,_complete_genome
NC_017671.1 Stenotrophomonas_maltophilia_D457,_complete_genome
NC_015947.1 Stenotrophomonas_maltophilia_JV3_chromosome,_complete_genome
NC_010943.1 Stenotrophomonas_maltophilia_K279a_chromosome,_complete_genome
NC_011071.1 Stenotrophomonas_maltophilia_R551-3_chromosome,_complete_genome
NC_022349.1 Vibrio_alginolyticus_NBRC_15630_=_ATCC_17749_chromosome_1,_complete_sequence
NC_022359.1 Vibrio_alginolyticus_NBRC_15630_=_ATCC_17749_chromosome_2,_complete_sequence
NC_015637.1 Vibrio_anguillarum_775_chromosome_II,_complete_sequence
**NC_022270.1 Vibrio_campbellii_ATCC_BAA-1116_chromosome_II,_complete_sequence**
NC_016628.1 Vibrio_furnissii_NCTC_11218_chromosome_2,_complete_sequence
NC_009784.1 Vibrio_harveyi_ATCC_BAA-1116_chromosome_II,_complete_sequence
**NC_022543.1 Vibrio_nigripulchritudo_str._SFn1_chromosome,_complete_genome**
NC_019971.1 Vibrio_parahaemolyticus_BB22OP_chromosome_2,_complete_sequence
NC_021822.1 Vibrio_parahaemolyticus_O1_K33_str._CDC_K4557_chromosome_II,_complete_sequence
NC_021821.1 Vibrio_parahaemolyticus_O1_Kuk_str._FDA_R31_chromosome_II,_complete_sequence
NC_004605.1 Vibrio_parahaemolyticus_RIMD_2210633_chromosome_2,_complete_sequence
NC_013457.1 Vibrio_sp._Ex25_chromosome_2,_complete_genome
NC_011744.2 Vibrio_splendidus_LGP32_chromosome_2,_complete_sequence
NC_004460.2 Vibrio_vulnificus_CMCP6_chromosome_II,_complete_sequence
NC_014966.1 Vibrio_vulnificus_MO6-24/O_chromosome_II,_complete_sequence
NC_005140.1 Vibrio_vulnificus_YJ016_chromosome_II,_complete_sequence
NC_013892.1 Xenorhabdus_bovienii_SS-2004_chromosome,_complete_genome
